# Intelligent Fault Diagnosis of Rolling-Element Bearings Using a Self-Adaptive Hierarchical Multiscale Fuzzy Entropy

**DOI:** 10.3390/e23091128

**Published:** 2021-08-30

**Authors:** Xiaoan Yan, Yadong Xu, Minping Jia

**Affiliations:** 1 School of Mechatronics Engineering, Nanjing Forestry University, Nanjing 210037, China; 2 School of Mechanical Engineering, Southeast University, Nanjing 211189, China; ydxu@seu.edu.cn (Y.X.); mpjia@seu.edu.cn (M.J.)

**Keywords:** hierarchical multiscale fuzzy entropy, bird swarm algorithm, support matrix machine, rolling element bearing, fault diagnosis

## Abstract

The fuzzy-entropy-based complexity metric approach has achieved fruitful results in bearing fault diagnosis. However, traditional hierarchical fuzzy entropy (HFE) and multiscale fuzzy entropy (MFE) only excavate bearing fault information on different levels or scales, but do not consider bearing fault information on both multiple layers and multiple scales at the same time, thus easily resulting in incomplete fault information extraction and low-rise identification accuracy. Besides, the key parameters of most existing entropy-based complexity metric methods are selected based on specialist experience, which indicates that they lack self-adaptation. To address these problems, this paper proposes a new intelligent bearing fault diagnosis method based on self-adaptive hierarchical multiscale fuzzy entropy. On the one hand, by integrating the merits of HFE and MFE, a novel complexity metric method, named hierarchical multiscale fuzzy entropy (HMFE), is presented to extract a multidimensional feature matrix of the original bearing vibration signal, where the important parameters of HMFE are automatically determined by using the bird swarm algorithm (BSA). On the other hand, a nonlinear feature matrix classifier with strong robustness, known as support matrix machine (SMM), is introduced for learning the discriminant fault information directly from the extracted multidimensional feature matrix and automatically identifying different bearing health conditions. Two experimental results on bearing fault diagnosis show that the proposed method can obtain average identification accuracies of 99.92% and 99.83%, respectively, which are higher those of several representative entropies reported by this paper. Moreover, in the two experiments, the standard deviations of identification accuracy of the proposed method were, respectively, 0.1687 and 0.2705, which are also greater than those of the comparison methods mentioned in this paper. The effectiveness and superiority of the proposed method are verified by the experimental results.

## 1. Introduction

The rolling-element bearing is one of the essential parts of rotating machinery; its health condition directly affects the safe and steady operation of mechanical equipment [1]. When bearing failure occurs, it can easily cause economic losses for enterprises and even catastrophic accidents. Besides, the properties of the fault-bearing vibration signal are usually nonlinear and nonstationary, which indicates that it is difficult to obtain helpful bearing fault information using traditional methods [2,3,4]. Therefore, exploring a new and effective fault feature extraction method to ensure the normal running of rolling-element bearings is of great significance.

Currently, many signal complexity metric methods have been proposed for describing the complexity and irregularity of bearing vibration signals and extracting the corresponding bearing fault feature information, including correlation dimension [5], Lempel–Ziv complexity [6], pattern spectrum entropy [7], approximate entropy [8], sample entropy (SE) [9], permutation entropy (PE) [10], symbol dynamic entropy (SDE) [11], dispersion entropy (DE) [12], fuzzy entropy (FE) [13], and so on. These methods have received great attention in the field of bearing fault diagnosis and have obtained many achievements. However, they only excavate bearing fault information on a single scale. Concerning this problem, based on single-scale signal complexity metric methods, many multiscale analysis methods (e.g., multiscale approximate entropy [14], multiscale sample entropy (MSE) [15], multiscale permutation entropy (MPE) [16,17], multiscale symbol dynamic entropy (MSDE) [18], multiscale dispersion entropy (MDE) [19], and multiscale fuzzy entropy (MFE) [20]) are presented for bearing fault diagnosis. For instance, Han et al. [21] adopted hierarchical Lempel–Ziv complexity to extract the fault information hidden in both low-frequency and high-frequency components and achieve intelligent fault diagnosis of rotating machinery. Yan et al. [22] used multiscale pattern gradient spectrum entropy (MPGSE) to extract bearing fault information, then extreme learning machine (ELM) to classify different bearing fault patterns. Gao et al. [23] combined the improved local mean decomposition (LMD), multiscale permutation entropy (MPE), and hidden Markov model (HMM) for identifying different fault types of rolling element bearings. Wu et al. [24] utilized MPE to obtain bearing fault features, then used support vector machine (SVM) to finish the fault identification of a rolling bearing. Gan et al. [25] presented a composite multiscale fluctuation dispersion entropy (CMFDE) to extract multiscale fault information of rolling bearings and realized a classification process for bearing fault types. Zheng et al. [26] proposed a generalized composite multiscale permutation entropy (GCMPE) to extract bearing fault information, and then used SVM to identify bearing fault types. Wang et al. [27] adopted the generalized refined composite multiscale sample entropy (GRCMSE) to extract bearing fault feature information, and then, the optimized support vector machine was used to finish bearing fault diagnosis. Tang et al. [28] proposed a hierarchical instantaneous energy density dispersion entropy (HIEDDE) to extract fault features, then the extracted features were regarded as the input of dynamic time warping (DTW) to automatically identify the fault types for a gearbox. Li et al. [29] proposed hierarchical fuzzy entropy (HFE) to extract bearing fault features and adopted an improved support-vector-machine-based binary tree to identify bearing fault patterns. Meanwhile, Li et al. [30] combined the local mean decomposition (LMD) and improved multiscale fuzzy entropy (IMFE) to complete intelligent fault diagnosis of a rolling bearing. However, there are two shortcomings in the above-reported methods. On the one hand, the above-mentioned methods can only excavate bearing fault feature information on the different scales or different levels of the original vibration signal, which indicates that multiscale fault features of different levels or different frequency bands of the original vibration signal are not taken into account simultaneously. In other words, the fault feature information obtained by the above-mentioned methods is not very comprehensive and rich. On the other hand, the above-mentioned methods rely on human experience to select the important parameters of entropy, so they are not adaptive, and this can easily affect the performance of fault feature extraction. Hence, to solve these issues, this paper proposes a novel signal complexity metric method named hierarchical multiscale fuzzy entropy (HMFE). Specifically, the proposed HMFE method consists of three modules (i.e., hierarchical decomposition, improved coarse-grained process, and fuzzy entropy calculation). Firstly, the hierarchical decomposition process of the collected original signal is conducted to obtain a series of hierarchical components. Subsequently, the improved coarse-grained process of each hierarchical component is carried out to effectively obtain the improved coarse-grained time series of each hierarchical component at different scales. Finally, the fuzzy entropy of each improved coarse-grained series is calculated to obtain the final hierarchical multiscale fuzzy entropy (it is equivalent to a feature matrix at different levels and scales). Besides, an effective intelligent optimizer named bird swarm algorithm (BSA) is employed to self-adaptively determine the parameters of HMFE. In summary, the contributions and novelties of this paper are summarized as follows:

(1) A novel signal complexity metric method named hierarchical multiscale fuzzy entropy (HMFE) is proposed via integrating hierarchic analysis into multiscale fuzzy entropy, which can effectively obtain more comprehensive and richer bearing fault feature information.
(2) The important parameters of HMFE are selected automatically by using the bird swarm algorithm (BSA) method, which can effectively avoid the disadvantages of manual selecting of the important parameters of the existing entropy.
(3) An intelligent bearing fault diagnosis method based on HMFE is presented, which can improve the identification accuracy of different health conditions of rolling-element bearings.
(4) Two experimental cases and comparative analysis are conducted to verify the effectiveness and superiority of the proposed method.

The organization of the rest of this paper is as follows. Section 2 introduces the self-adaptive hierarchical multiscale fuzzy entropy, including the basic theory, adaptive parameter selection, and simulation analysis of HMFE. In Section 3, the concept of the support matrix machine is summarized. Section 4 shows the flowchart of the proposed method for bearing fault diagnosis. Section 5 validates the effectiveness and superiority of the proposed method through two experimental examples and contrastive analysis. The conclusions are drawn in Section 6.

## 2. Self-Adaptive Hierarchical Multiscale Fuzzy Entropy

In this section, self-adaptive hierarchical multiscale fuzzy entropy, which is mainly composed of HMFE and its adaptive parameter selection, is introduced.

### 2.1. HMFE

**Figure 1:**
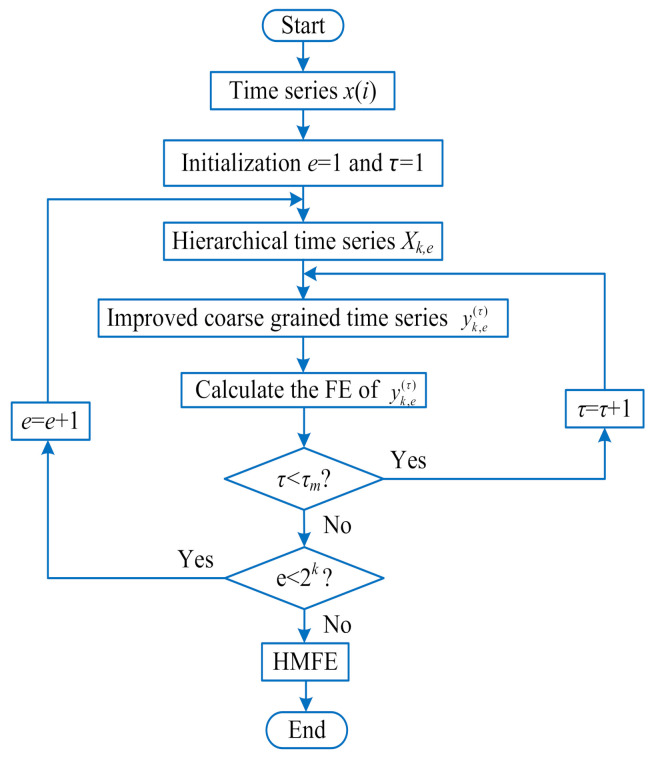
Flowchart of the HMFE method.

In this section, based on the advantages of HFE and MFE, a novel signal complexity metric method named hierarchical multi-scale fuzzy entropy (HMFE) is proposed. Figure 1 shows the flowchart of the proposed HMFE method. For a given time series {x(i),i=1,2,⋯,N}, the calculation process of HMFE is described in detail as follows:

(1) Firstly, the average and difference operator are respectively defined as follows:
  (1) $$Q_{0}(x)=\frac{x(2i)+x(2i+1)}{2},i=0,1,2,\cdots ,2^{n-1}$$
  (2) $$Q_{1}(x)=\frac{x(2i)-x(2i+1)}{2},i=0,1,2,\cdots ,2^{n-1}$$
  where *n* denotes the positive integer, $2^{n-1}$ represents the length of the two operators, and $Q_{0}(x)$ and $Q_{1}(x)$ represent the low-frequency and high-frequency components of the original time series in the first layer decomposition, respectively.
(2) Then, to implement the hierarchical analysis of a time series, when *j* = 0 or 1, the matrix form of the *k*-th layer operator $Q_{j}^{k}$ is expressed as:
  (3) $$Q_{j}^{k}={\left[\begin{array}{ccccccc} \frac{1}{2} & \frac{{\left(-1\right)}^{j}}{2} & 0 & 0 & \cdots & 0 & 0\\ 0 & 0 & \frac{1}{2} & \frac{{\left(-1\right)}^{j}}{2} & \cdots & 0 & 0\\ 0 & 0 & 0 & 0 & \cdots & \frac{1}{2} & \frac{{\left(-1\right)}^{j}}{2} \end{array}\right]}_{2^{n-1}\times 2^{n}}$$
(3) To obtain the hierarchical components $X_{k,e}$ of each layer in the process of hierarchical decomposition, here we define a one-dimensional vector as $\left[\gamma_{1},\gamma_{2},\cdots ,\gamma_{k}\right]$ and an integral value as $e=\sum_{p=1}^{k}2^{k-p}\gamma_{p}$, where $\left\{\gamma_{p},p=1,2,\cdots ,k\}\in \{0,1\right\}$ represents the average or difference operator at the *p*-th layer. Accordingly, the hierarchical component of the *e*-th node in the *k*-th layer can be expressed as:
  (4) $$X_{k,e}=Q_{\gamma_{k}}^{k}\cdot Q_{\gamma_{k-1}}^{k-1}\cdot \cdots \cdot Q_{\gamma_{1}}^{1}\cdot x$$
  where *x* represents the given time series.
(4) Next, the improved coarse-grained time series $y_{k,e}^{\left(\tau \right)}=\left\{y_{k,e,1}^{\left(\tau \right)},y_{k,e,2}^{\left(\tau \right)},\cdots \right\}$ of each hierarchical component at the τ scale factor can be calculated by
  (5) $$y_{k,e,j}^{\left(\tau \right)}=\frac{1}{\tau }\sum_{i=j}^{j+\tau -1}X_{k,e}^{i},1\leq j\leq N-\tau +1$$
  where *N* represents the length of the given time series *x.*
(5) According to the definition of fuzzy entropy, calculate the fuzzy entropy of each improved coarse-grained series $y_{k,e}^{\left(\tau \right)}$, so the final hierarchical multiscale fuzzy entropy can be obtained by the following:
  (6) $$HMFE(x,m,k,e,\tau ,r)=FE(y_{k,e}^{\left(\tau \right)},m,\tau ,r)$$
  where FE(·) represents the fuzzy entropy operation, *m* is the embedded dimension, *k* is the decomposition level, *e* represents the hierarchical node, τ denotes the scale factor, r=0.15×SD is the similarity tolerance controlling the width of membership function, and *SD* is the standard deviation of the original time series. In Figure 1, $\tau_{m}$ represents the predefined largest scale factor.

### 2.2. Adaptive Parameter Selection of HMFE

**Figure 2:**
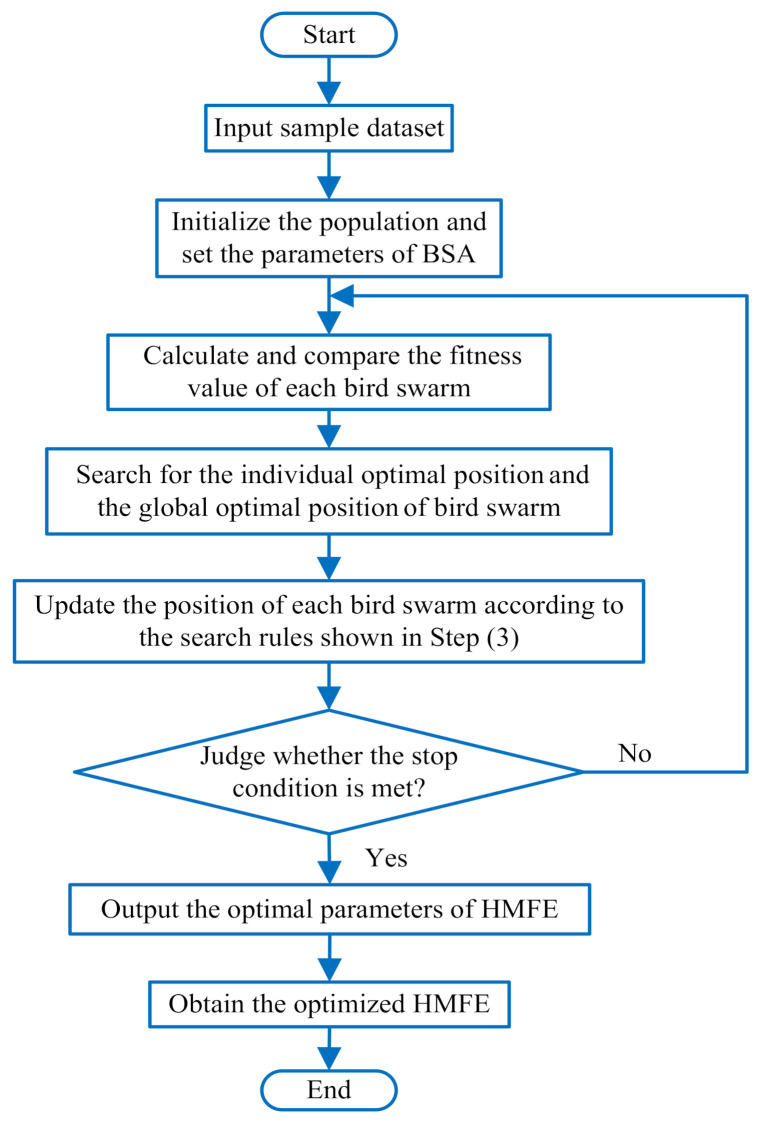
Flowchart of parameter optimization of HMFE.

The previous studies have shown that fault feature extraction performance of the existing entropies (e.g., MSE, MPE, MFE, and HFE) is greatly affected by their parameter settings. As with traditional entropies (e.g., MSE, MPE, MFE, and HFE), the parameters of HMFE have a great impact on its performance, which indicates that it is necessary to adopt an effective method to achieve adaptive selection of the parameters of HMFE. At present, the intelligent swarm optimization algorithm has been proven to be effective in solving parameter selection problems, including particle swarm optimization (PSO), ant colony algorithm (ACO), bat algorithm (BA), and so on. Bird swarm algorithm (BSA) is a new bionic optimization algorithm proposed by Meng et al. [31] in 2016. Compared with other optimization algorithms, BSA has the advantages of high accuracy, strong stability, and fast convergence in the parameter optimization. Therefore, in this paper, BSA is adopted to automatically select several important parameters (i.e., the embedding dimension *m*, the decomposition level *k* and the scale factor τ) of HMFE. Figure 2 plots the flowchart of parameter optimization of HMFE. The specific process of parameter optimization of HMFE based on BSA method is summarized as follows:

(1) Initialize the population and set the BSA parameters. When the number of iterations *t* = 0, set the bird swarm size to *N* = 30 and the maximum iteration number to *M* = 50, initialize the flight frequency *FQ*, foraging frequency *P*, and several constants (i.e., *C*, *S*, *FL*, *a*_1_ and *a*_2_).
(2) Calculate and compare the fitness value. According to the fitness function shown in Equation (7), the fitness value of bird swarm is calculated and compared to determine the optimal position of the individual and whole bird swarm.
  (7) $$\mathrm{fitness}(i)=1-\frac{x_{i}}{x_{c}+x_{i}}$$
  where *x_i_* is the number of misclassified samples, *x_c_* is the number of samples correctly classified, and fitness(*i*) is the current fitness value of the *i*-th bird. When fitness(*i*) achieves the maximum value, *p_i,j_* is the corresponding optimal position of the individual bird swarm, and *g_j_* is the corresponding optimal position of the whole bird swarm.
(3) The iterative operation is performed repeatedly, and the position update formula is determined by judging whether the operation *t*% × *FQ* has a remainder. Specific rules are summarized as follows:

If there is a remainder for *t*% × *FQ*, a uniformly distributed number is randomly generated. When the random number is less than the foraging frequency *P*, the foraging behavior is performed, and Equation (8) is used to update the position. Otherwise, the sentinel behavior is performed, and Equation (9) is used to update the position.

(8) $$\begin{array}{c} x_{i,j}^{t+1}=x_{i,j}^{t}+(p_{i,j}-x_{i,j}^{t})\times C\times \mathrm{rand}(0,1)+\\ \left(g_{j}-x_{i,j}^{t})\times S\times \mathrm{rand}(0,1\right) \end{array}$$

where rand(0,1) is a uniformly distributed random number between 0 and 1, *p_i,j_* is the current best position of the *i*-th bird, *g_j_* is the current best position of bird swarm, and *C* and *S* are two positive numbers, which are called the cognitive acceleration coefficient and social acceleration coefficient, respectively.

(9) $$\left\{\begin{array}{l} x_{i,j}^{t+1}=x_{i,j}^{t}+A_{1}({\mathrm{mean}}_{j}-x_{i,j}^{t})\times \mathrm{rand}(0,1)+\\ \;A_{2}(p_{k,j}-x_{i,j}^{t})\times \mathrm{rand}(-1,1)\\ A_{1}=a_{1}\times \exp(-\frac{{\mathrm{pfit}}_{i}}{\mathrm{sumfit}+\varepsilon }\times N)\\ A_{2}=a_{2}\times \exp(\left(\frac{{\mathrm{pfit}}_{i}-{\mathrm{pfit}}_{k}}{\left|{\mathrm{pfit}}_{k}-{\mathrm{pfit}}_{i}\right|+\varepsilon }\right)\frac{N\times {\mathrm{pfit}}_{k}}{\mathrm{sumfit}+\varepsilon }) \end{array}\right.$$

where $x_{i,j}^{t}$ is the position of individual birds in the *t*-th iteration, $x_{i,j}^{t+1}$ is the position of individual birds in the *t* + 1 iteration, $a_{1}$ and $a_{2}$ are the positive number between 0 and 2, ${\mathrm{pfit}}_{i}$ is the optimal fitness value of the *i*-th bird, sumfit is the sum of the optimal fitness values of bird swarm, ε is the smallest constant that avoids dividing the denominator by zero, k(k≠i) is a positive integer between 1 and *N*, and ${\mathrm{mean}}_{j}$ is the *j*-th element of the average position of the entire bird swarm.

If there is no remainder for *t*% × *FQ* when individual birds are the producers, Equation (10) is used to update the position. When individual birds are beggars, Equation (11) is used to update the position.

(10) $$x_{i,j}^{t+1}=x_{i,j}^{t}+\mathrm{randn}(0,1)\times x_{i,j}^{t}$$

(11) $$x_{i,j}^{t+1}=x_{i,j}^{t}+(x_{k,j}^{t}-x_{i,j}^{t})\times FL\times \mathrm{randn}(0,1)$$

where $x_{i,j}^{t}$ is the position of individual birds in the t-th iteration; $x_{i,j}^{t+1}$ is the position of individual birds in the *t* + 1 iteration; randn(0,1) represents the random number of Gaussian distribution with mean value 0 and standard deviation 1, k∈[1,2,3,⋯,N],k≠i; and *FL* denotes the integer between 0 and 2.

(4) Update the position of each bird swarm according to the rules in step (3). If the individual of the current bird swarm is better than the individual of the previous bird swarm, the current individual bird swarm is regarded as the optimal position. Otherwise, the previous individual bird swarm is retained as the optimal position to continue the update of bird swarm.
(5) Judge whether the stop condition is met. If the maximum number of iterations or the minimum error rate is reached, the whole optimization process will be stopped, and the optimal position of bird swarm (i.e., the optimal combination parameters of HMFE) will be outputted. Otherwise, the iteration process will continue to be conducted until the stop condition is satisfied.

### 2.3. Comparison Analysis Using Simulation Signal

To investigate the performance of the proposed HMFE method, according to the literature [32], one bearing vibration signal $x_{OR}(t)$ (i.e., the simulation signal 1) containing outer race fault and one bearing vibration signal $x_{IR}(t)$ (i.e., the simulation signal 2) containing inner race fault are established by using Equations (12) and (13), respectively.

(12) $$\left\{\begin{array}{l} x_{OR}(t)=f_{1}(t)*h_{1}(t)+n(t)\\ f_{1}(t)=\sum_{k=0}^{N-1}\delta (t-kT_{1})\\ h_{1}(t)=e^{-600\pi t}\cos(2\pi f_{n}t) \end{array}\right.$$

(13) $$\left\{\begin{array}{l} x_{IR}(t)=f_{2}(t)*h_{2}(t)+n(t)\\ f_{2}(t)=\sum_{k=0}^{N-1}\cos(2\pi f_{r}t)\delta (t-kT_{2})\\ h_{2}(t)=e^{-800\pi t}\cos(2\pi {\hat{f}}_{n}t) \end{array}\right.$$

where the asterisk * indicates the convolution operation; $f_{1}(t)$ and $f_{2}(t)$ are, respectively, the cyclic impact signal caused by bearing outer race fault and inner race fault; *n*(*t*) is the white Gaussian noise with a mean of 0 and standard deviation of 1; δ(⋅) indicates the Dirac delta function, k=0,1,⋯,N−1; *N* indicates the total number of impact impulses; and $T_{1}$ and $T_{2}$ are the time intervals between two adjacent impulses under bearing outer race fault and inner race fault, respectively. Bearing outer race fault frequency and inner race fault frequency are $f_{OR}=1/T_{1}$ = 50 Hz and $f_{IR}=1/T_{2}$ = 90 Hz, respectively. $f_{r}$ = 25 Hz is the rotation frequency of the driving shaft, $h_{1}(t)$ and $h_{2}(t)$ are, respectively, the impulse response signal caused by the bearing outer race fault and that caused by the inner race fault; and $f_{n}$ = 3000 Hz and ${\hat{f}}_{n}$ = 5000 Hz are the natural frequencies of the excitation system under bearing outer race fault and inner race fault, respectively. The sampling frequency and data length of simulation signal are set as 16,384 Hz and 4096 points, respectively.

**Figure 3:**
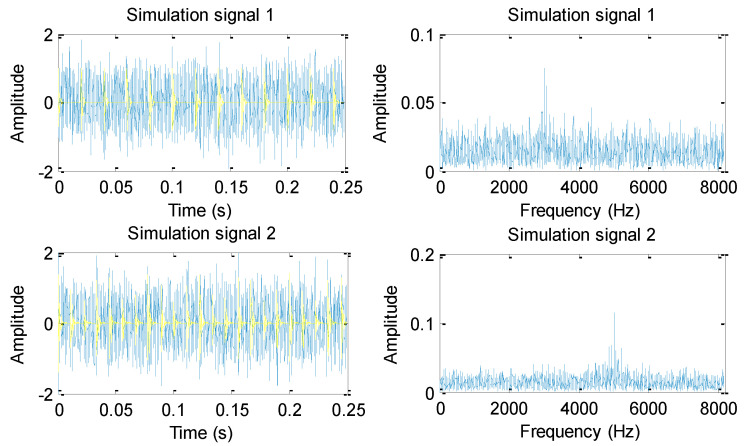
Time domain waveforms and their corresponding amplitude spectra for two simulation signals.

**Figure 4:**
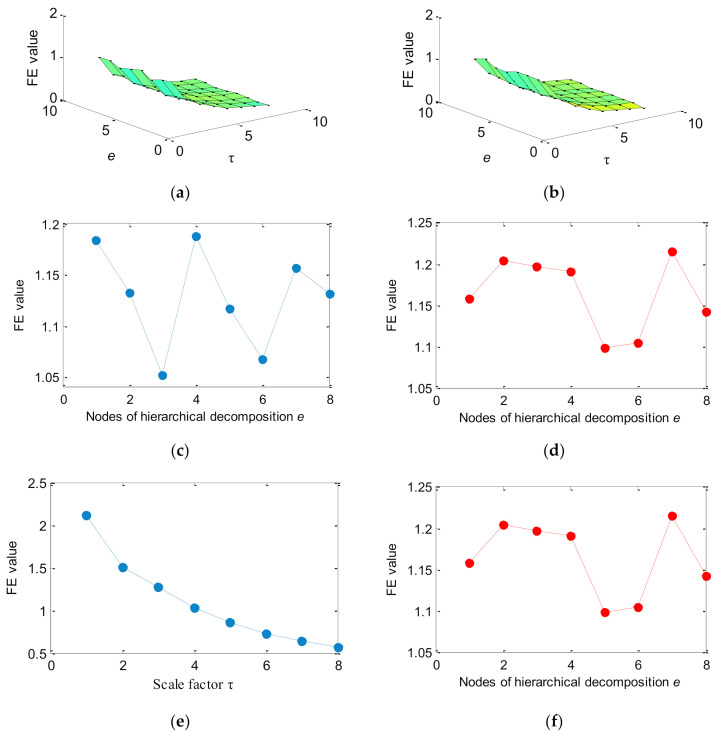
FE value obtained by different methods for two simulation signals: (**a**) HMFE of simulation signal 1, (**b**) HMFE of simulation signal 2, (**c**) HFE of simulation signal 1, (**d**) HFE of simulation signal 2, (**e**) MFE of simulation signal 1, and (**f**) MFE of simulation signal 2.

Figure 3 shows the time domain waveforms and their corresponding amplitude spectra for two simulation signals. As can be seen from Figure 3, the periodic impact impulses of two simulation signals are drowned in the random noise. That is, it is difficult to identify bearing fault types by the direct observation of time domain waveform, which means that an effective method is needed to extract the fault features of the two simulation signals for distinguishing and recognizing them. Hence, the Euclidean distance (ED) of three entropies (i.e., HMFE, HFE, and MFE) of two simulation signals are calculated to compare their feature extraction ability. In the three methods (i.e., HMFE, HFE, and MFE), the embedded dimension is *m =* 3 and the similarity tolerance r=0.15×SD, where *SD* is the standard deviation of the simulation signal. Besides, for HMFE and HFE, the decomposition level is *k =* 3. For HMFE and MFE, the scale factor is τ = 8. Figure 4a–d shows the FE value obtained by the three methods (i.e., HMFE, HFE, and MFE) for two simulation signals. Apparently, the FE value at different levels and scales can be extracted by using HMFE, whereas HFE and MFE can only obtain the FE value of different levels or scales. This indicates that HMFE can obtain more comprehensive feature information compared to HFE and MFE. Table 1 lists the Euclidean distance (ED) and calculation time of different methods. As shown in Table 1, the ED of the proposed HMFE is largest, which shows that the feature information obtained by HMFE is more differentiated compared with HFE and MFE. Besides, the calculation time of HMFE is significantly less than that of MFE, but it is greater than that of HFE. This is mainly due to the hierarchical decomposition and multiscale analysis process being integrated in the HMFE method, which increases the computational burden of HMFE to some extent, but it is generally acceptable for practical bearing vibration signal analysis.

## 3. Support Matrix Machine

The intelligent fault identification step is required after the entropy-based fault feature extraction. At present, many linear or nonlinear classification models have been proposed for intelligent bearing fault identification, including linear discriminant analysis (LDA), BP neural network (BPNN), K-nearest neighbor (KNN), extreme learning machine (ELM) [33], partial least squares (PLS) [34], and support vector machine (SVM). However, these methods are only applicable to the classification of multidimensional feature vectors. When the above-mentioned classification model is used for the processing of the multidimensional feature matrix, their performance will be reduced. Hence, to solve this problem, a new nonlinear classification model named support matrix machine (SMM) was recently proposed by Luo et al. [35] in 2015, which can automatically learn the useful discriminant information from the multidimensional feature matrix. In view of this, in this paper, our plan is to adopt SMM to process the multidimensional feature matrix constructed by HMFE and achieve intelligent fault identification of the rolling bearing. The basic theory of SMM is described as follows:

Suppose that ${\left\{X_{i},y_{i}\right\}}_{i=1}^{n}$ is one given training set, where $X_{i}\in R^{d_{1}\times d_{2}}$ is the i-th input matrix, $y_{i}\in \{1,-1\}$ is the training label, and *d*_1_ and *d*_2_ represent the number of rows and columns of the input matrix, respectively. Simply speaking, SMM implements the model training and classification process through the hinge loss function and spectral elastic network penalty function, as shown below.

(14) $$\begin{array}{l} \underset{W,b}{\arg\;\min}\frac{1}{2}\mathrm{tr}(W^{T}W)+\lambda {\left\|W\right\|}_{*}+C\sum_{i=1}^{n}\xi_{i}\\ s.t.y_{i}[\mathrm{tr}(W^{T}X_{i})+b]\geq 1-\xi_{i},\forall i=1,2,\ldots ,n \end{array}$$

where $\mathrm{tr}(W^{T}W)={\left\|W\right\|}_{F}^{2}$ is the squared F-norm [36], $W\in R^{d_{1}\times d_{2}}$ is the regression coefficient matrix, ${\left\|W\right\|}_{*}$ is the kernel norm, λ and *C* are respectively kernel parameter and penalty parameter, and *b* is the bias term. Spectral elastic network penalty function consists of the squared F-norm and kernel norm. The squared F-norm can be used to control the complexity of model and prevent the over-fitting phenomenon. The kernel norm can approximately replace the rank of the regression coefficient matrix. Therefore, based on the classification property of spectral elastic networks, SMM can effectively capture intrinsic feature information and correlation in the input matrix. The alternating direction multiplier method (ADMM) can be used to optimize SMM. In particular, after introducing auxiliary variables $Z\in R^{d_{1}\times d_{2}}$, Equation (14) can be rewritten as:

(15) $$\begin{array}{l} \underset{W,b,Z}{\arg\;\min}P(W,b)+Q(Z)\\ s.t.\;Z-W=0 \end{array}$$

where P(W,b) and Q(Z) can be expressed as follows:

(16) $$\begin{array}{l} P(W,b)=\frac{1}{2}tr(W^{T}W)+C\sum_{i=1}^{n}{\left\{1-y_{i}[tr(W^{T}X_{i})+b]\right\}}_{+}\\ Q(Z)=\tau {\left\|Z\right\|}_{*} \end{array}$$

Specifically, Equation (14) can be solved by the augmented Lagrange multiplier method [37], that is:

(17) $$L(Z,W,b,M)=P(W,b)+Q(Z)+tr(M^{T}(Z-W))+\frac{\beta }{2}{\left\|\left(Z-W\right)\right\|}_{F}^{2}$$

where $M\in R^{d_{1}\times d_{2}}$ is the Lagrange multiplier and β is a positive hyperparameter. The updated equations of *Z*, *W*, *B* and the Lagrange multiplier *M* can be expressed as:

(18) $$\left\{\begin{array}{l} Z^{t+1}=\underset{Z}{\arg\;\min}L(Z,W^{t},b^{t},M^{t})\\ \left(W^{t+1},b^{t+1})=\underset{\left(W,b\right)}{\arg\;\min}L(Z^{t+1},W,b,M^{t}\right)\\ M^{t+1}=M^{t}-\beta (W^{t+1}-Z^{t+1}) \end{array}\right.$$

where *t* is the number of iterations. According to [35], the optimal solution of Equation (18) can be written as:

(19) $$\left\{\begin{array}{l} \hat{W}=\frac{1}{\beta +1}(M+\beta Z+\sum_{i=1}^{n}{\hat{\alpha }}_{i}y_{i}X_{i})\\ \hat{b}=\frac{1}{n}\sum_{i=1}^{n}\left\{y_{i}-\mathrm{tr}({\hat{W}}^{T}X_{i})\right\}\\ \hat{Z}=\frac{1}{\beta }D_{\tau }(\beta W-M) \end{array}\right.$$

Finally, the decision function of SMM can be expressed as:

(20) $$f(X)=\mathrm{sgn}(\mathrm{tr}(W^{T}X)+b)$$

where sgn(·) indicates the sign function and tr(·) indicates the trace of a matrix.

## 4. Proposed Method

**Figure 5:**
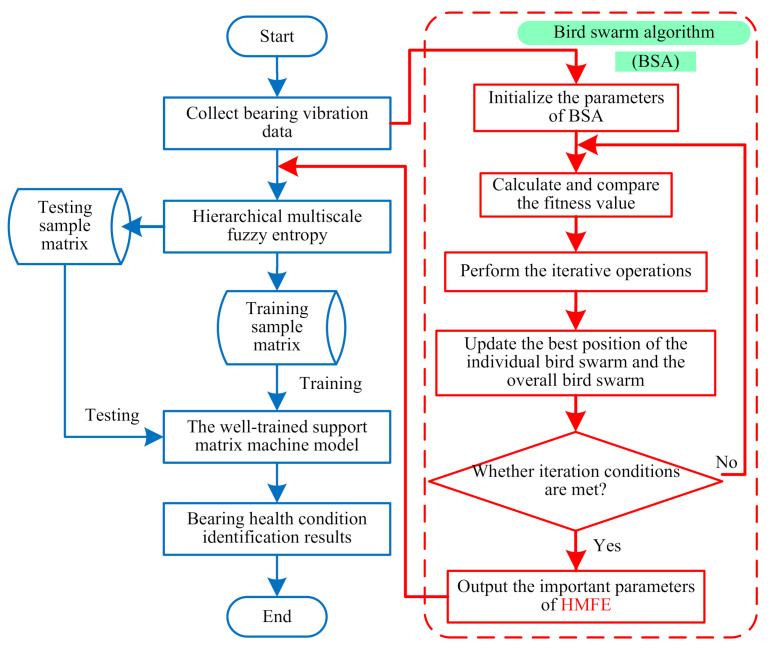
Flowchart of the proposed method for bearing fault identification.

To obtain abundant fault information and improve bearing fault identification accuracy, a new approach based on self-adaptive hierarchical multiscale fuzzy entropy is proposed for intelligent fault diagnosis of rolling-element bearings. The proposed method consists of three steps (i.e., bearing vibration data collection, hierarchical multiscale feature extraction, and bearing health condition identification). Figure 5 shows the flowchart of the proposed method, and its main steps are described below:

**Step1:***Bearing vibration data collection*. Bearing vibration data under different health conditions are obtained by installing the accelerometer on the experimental equipment.

**Step2:***Hierarchical multiscale feature extraction*. Using the HMFE method, the FE value of bearing vibration signals at different levels and scales are calculated, where the important parameters of the HMFE method are automatically determined by using the bird swarm algorithm method. Meanwhile, the calculated HMFE of each bearing health condition is adopted to construct a multidimensional feature matrix.

**Step3:***Bearing health condition identification*. The constructed multidimensional feature matrix is randomly divided into the training sample matrix and testing sample matrix, where the training sample matrix is adopted to train the SMM classification model, and the testing sample matrix is fed into the well-trained SMM classification model to automatically identify different bearing health conditions.

## 5. Experimental Verification

In this section, two experiments about bearing fault diagnosis are conducted to show the effectiveness of the proposed method. Furthermore, comparisons among the proposed method and several representative entropies are adopted to highlight the superiority of the proposed method. Finally, we discuss the future research direction.

### 5.1. Case 1: Bearing Benchmark Data from CWRU

**Figure 6:**
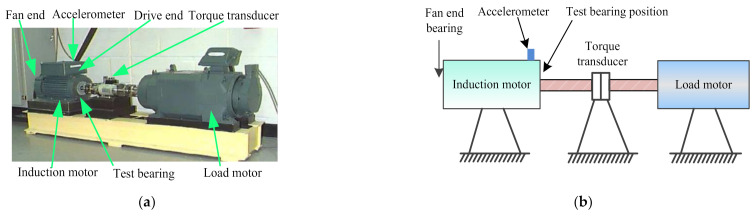
(**a**) The experimental platform and (**b**) its schematic diagram.

**Figure 7:**
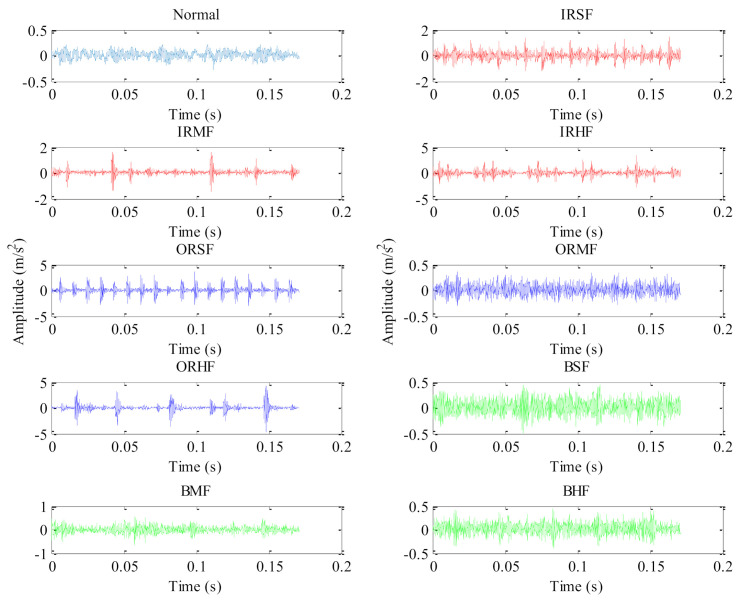
Time domain waveforms of bearing vibration data under different health conditions in case 1.

Bearing benchmark data from Case Western Reserve University (CWRU) is applied to validate the effectiveness of the proposed method. Figure 6 shows the experimental platform and its schematic diagram, which are mainly composed of induction motor, testing bearing, torque transducer, and load motor. Table 2 lists the specifications of the test bearing. In the experiment process, the sampling frequency is set as 12 kHz and the motor speed is set as 1797 rpm. Besides, nine single-point faults (i.e., inner race slight fault (IRSF), inner race medium fault (IRMF), inner race heavy fault (IRHF), outer race slight fault (ORSF), outer race medium fault (ORMF), outer race heavy fault (ORHF), ball slight fault (BSF), ball medium fault (BMF), and ball heavy fault (BHF)) are manufactured on normal bearings by using electric discharging machining technique [38]. One accelerometer is mounted on the bearing block of the drive-end of the induction motor to collect, respectively, bearing vibration data containing the ten health conditions (i.e., normal, IRSF, IRMF, IRHF, ORSF, ORMF, ORHF, BSF, BMF, and BHF). For each bearing health conditions, 50 data samples with a length of 2048 points are intercepted by using a non-overlapping sliding window. That is, a total of 500 data samples are obtained for the whole health conditions, where 25 data samples of each bearing health condition are randomly selected as the training set and the remaining 25 data samples are treated as the testing set. Table 3 presents a detailed description of bearing vibration data under ten health conditions. Figure 7 shows the time domain waveform of bearing vibration data under different health conditions in case 1. Seen from Figure 7, due to the measured bearing vibration data containing some background noises and having nonlinear non-stationary characteristics, it is difficult to accurately distinguish different health states of bearings by directly observing the time domain waveforms.

**Figure 8:**
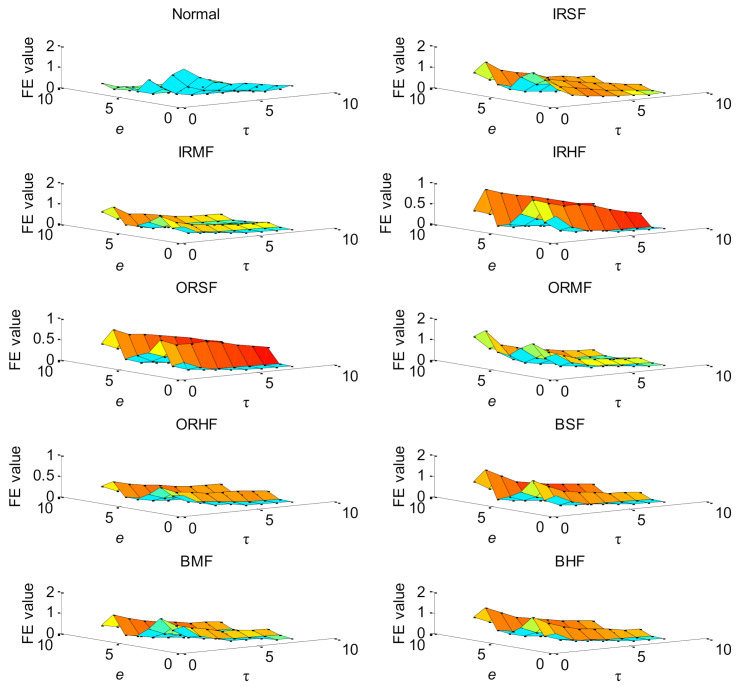
HMFE of bearing vibration data under different health conditions in case 1.

**Figure 9:**
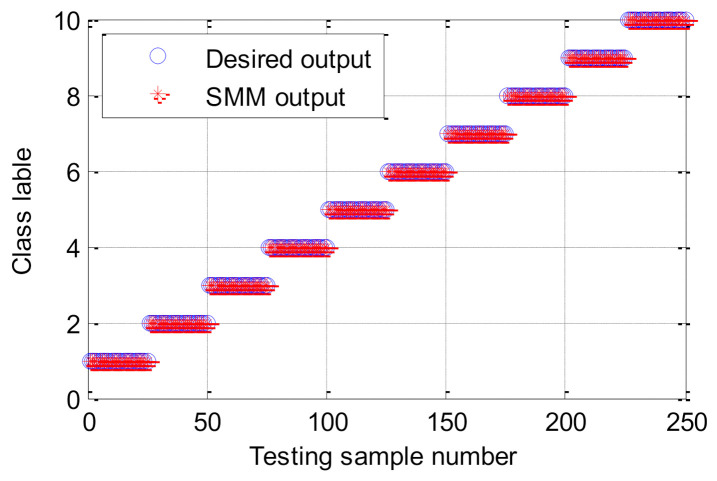
Classification results of the proposed method in case 1.

In order to effectively extract bearing fault features and improve the identification accuracy, the proposed method is adopted to analyze the experimental data listed in Table 3. Firstly, the BSA method is used to automatically determine the optimal parameters of HMFE at the embedded dimension *m* = 3, the decomposition level *k* = 3, and the scale factor *τ* = 8. Subsequently, the HMFE method with the optimal parameters is conducted to calculate the FE value of different bearing health conditions at different levels and scales. Meanwhile, the calculated HMFE of each bearing health condition is adopted to build the multidimensional feature matrix with a size of 8 × 8 × 500. Figure 8 shows the calculation results of HMFE of bearing vibration data under different health conditions. It can be seen from Figure 8 that the FE value of different bearing health conditions is different at some levels or scales due to the proper integration of hierarchical decomposition and multiscale coarse-grained analysis, which helps for the subsequent identification of bearing health conditions. Finally, the constructed multidimensional feature matrix is randomly divided into the training sample matrix with a size of 8 × 8 × 250 and the testing sample matrix with a size of 8 × 8 × 250. Besides, the SMM classification model is trained by the training sample matrix, and the testing sample matrix is fed into the well-trained SMM model to identify different bearing health conditions and automatically report the fault diagnosis results. Figure 9 shows the identification results of the proposed method in the first trial. Seen from Figure 9, the proposed method can achieve identification accuracy of 100%, which means that the proposed method is effective in identifying different fault categories and severities of rolling-element bearings.

To show the effectiveness of the parameter optimization process used in the proposed HMFE, the fault identification accuracy of the proposed method containing different parameters of HMFE is calculated, and the results are shown in Table 4. Seen from Table 4, when the BSA method is used to select the optimal parameters (i.e., the embedded dimension *m* = 3, the decomposition level *k* = 3, and the scale factor *τ* = 8) of HMFE, the proposed method with the parameter optimization process can obtain a high identification accuracy. This means that the BSA-parameter optimization process in the proposed HMFE is very useful for bearing fault identification. Besides, as shown in Table 4, the identification accuracy is greater than 95% only when the scale factor *τ* is higher than or equal to 7. For the phenomenon of the lower accuracy at low scale factor *τ*, here an explanation is given. Concretely, in the HMFE method, when the embedded dimension *m* and decomposition level *k* stay constant, if the scale factor *τ* is set as smaller, the feature matrix with a smaller dimension will be extracted, which indicates that the feature information obtained by HMFE is relatively less and the identification accuracy of the proposed method is reduced. Theoretically, the larger scale factor *τ* has more feature information and higher identification accuracy. However, the bigger the scale factor *τ* is, the longer the calculation time of the proposed method is, and the extracted features will contain some redundant information. Hence, regarding the scale factor *τ,* bigger is not necessarily better, and it needs to be selected appropriately.

**Figure 10:**
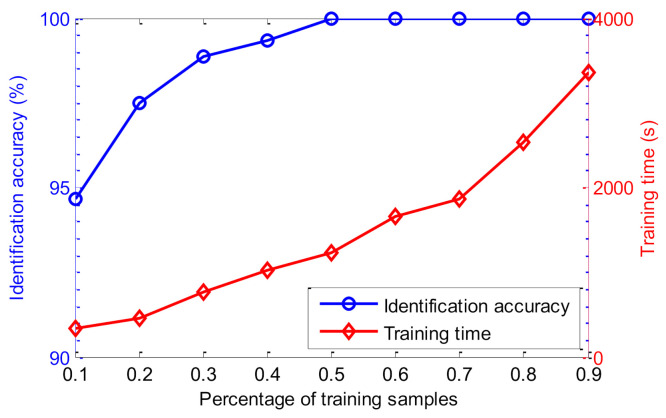
The identification results of the proposed method under different percentages of training samples in case 1.

Due to the number of training and testing samples having a great influence on the identification performance of the proposed method, the identification results of the proposed method are further investigated at different percentages of training samples. Specifically, identification results of the proposed method are calculated with the number of training samples set at, respectively, 50, 100, 150, 200, 250, 300, 350, 400, and 450. Identification accuracy and training time of the proposed method are shown in Figure 10 for when the percentage of training samples in the whole dataset is, respectively, 10% (50/500), 20% (100/500), 30% (150/500), 40% (200/500), 50% (250/500), 60% (300/500), 70% (350/500), 80% (400/500), and 90% (450/500). As can be seen from Figure 10, when the percentage of training samples is equal to or greater than 0.5 (i.e., 50%), the proposed method achieves an identification accuracy of 100%. However, as the percentage of training samples increases, the training time of the proposed method has an upward trend. In other words, the higher the number of training samples, the better trained the SMM model is, but the training time of the proposed method will be longer. Therefore, to strike a balance between identification accuracy and training time, the number of training and testing samples is set to the same percentages used in this paper.

To further show the effectiveness of the SMM classification model used in the proposed method, the proposed HMFE is combined with four classification models (i.e., SMM, SVM, ELM, and BPNN) to identify bearing fault patterns. The parameters of these classification models are set based on the previous work [39]. Specifically, in the SMM and SVM classifier, the kernel parameter *λ* =1/*n* and the penalty parameter *C =* 1, where *n* represents the dimensions of the extracted feature matrix. In the ELM classifier, the activation function uses a Sigmoid function, and the number of hidden nodes is *N* = 20. In the BPNN classifier, the number of hidden nodes is *N* = 20, the maximum training number is *I* = 500, and the learning rate is *σ* = 0.1. Each method is conducted for 5 trials to compare the results objectively. Table 5 lists the identification results of combining HMFE and different classification models (i.e., SMM, SVM, ELM, and BPNN). It can be clearly seen from Table 5 that the average recognition accuracy of the proposed method (i.e., the combination of HMFE and SMM) is significantly higher than the other combination methods, which indicates that the validity of using SMM classification model in the proposed method is verified.

**Figure 11:**
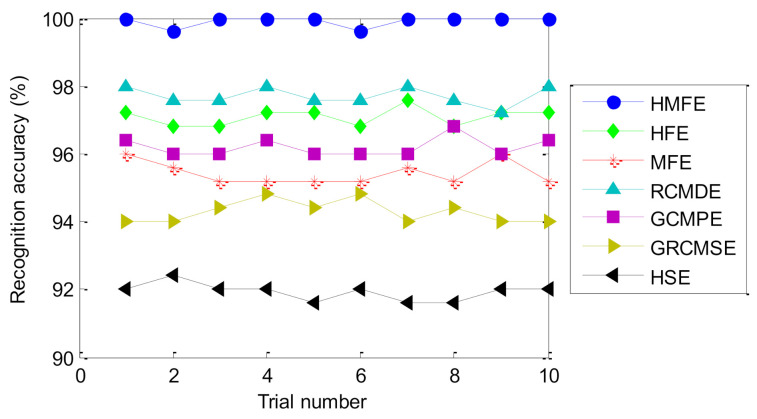
The identification results of 10 trials of different methods in case 1.

To show the superiority of the proposed method, comparisons are made between the proposed method and six existing representative entropies (i.e., GCMPE [26], GRCMSE [27], HFE [29], MFE [30], refined composite multiscale dispersion entropy (RCMDE) [40], and hierarchical sample entropy (HSE) [41]). To avoid the randomness and occasionality of the recognition results of different methods, 10 trials of each method are conducted to observe and compare the identification results. Besides, to ensure a fair comparison, the important parameters of all methods are selected by the BSA method, and the classification process is completed by the SMM model. Table 6 lists the setting of the optimal parameters of different methods. Figure 11 shows the identification results of different methods in 10 trials. Besides, Table 7 gives the final fault diagnosis results of the different methods for 10 trials, including the maximum accuracy, minimum accuracy, average accuracy, and standard deviation. As shown in Figure 11 and Table 7, the average identification accuracy of the proposed method is 99.92%, which is higher than that of the six comparative methods (i.e., HFE, MFE, RCMDE, GCMPE, GRCMSE, and HSE), which are 97.08%, 95.44%, 97.72%, 96.20%, 94.28%, and 91.92%, respectively. This indicates that the identification ability of the proposed method for bearing health condition is better than the other methods. Moreover, the standard deviation of the proposed method is 0.1687, which is smaller than that of the six comparative methods (i.e., HFE, MFE, RCMDE, GCMPE, GRCMSE and HSE), which are 0.2700, 0.3373, 0.2700, 0.2828, 0.3293, and 0.2530, respectively. That is, compared with the six comparative methods (i.e., HFE, MFE, RCMDE, GCMPE, GRCMSE, and HSE), the proposed method has a better stability in identifying bearing fault category and severity. Hence, the effectiveness and advantages of the proposed method have been verified in the comparative analysis of case 1.

### 5.2. Case 2: Bearing Vibration Data from Laboratory

**Figure 12:**
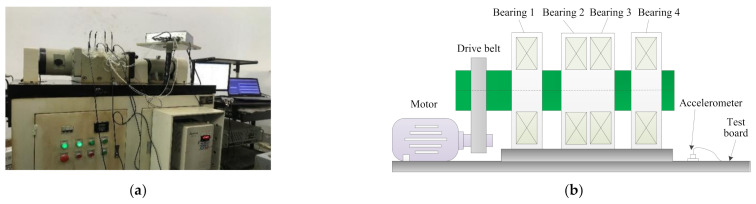
(**a**) Bearing accelerated life test bench and (**b**) its corresponding schematic structure drawing.

**Figure 13:**
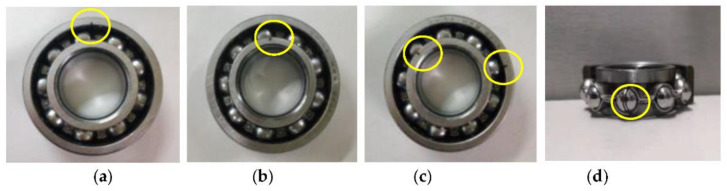
Photo of the faulty bearing: (**a**) ORF, (**b**) IRF, (**c**) OIRCF, and (**d**) BF.

**Figure 14:**
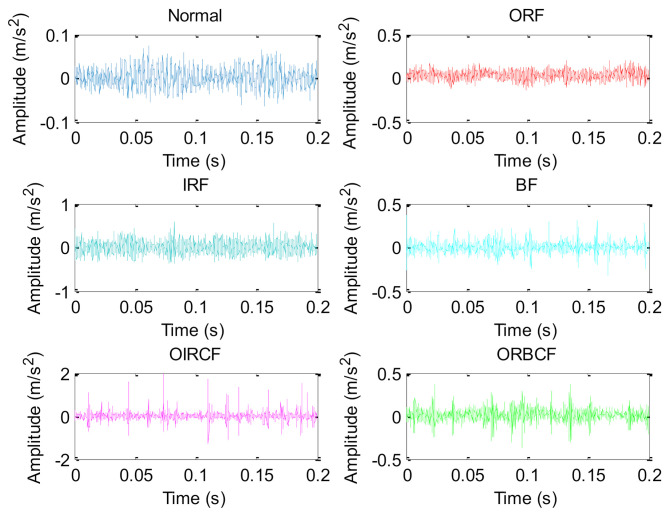
Time domain waveforms of bearing vibration data under different health conditions in case 2.

In this section, bearing vibration data collected from an ABLT-1A experimental device is used to verify the effectiveness of the proposed method in identifying bearing fault patterns. Figure 12a,b show, respectively, the bearing accelerated life test bench and its corresponding structure diagram, which is mainly composed of motor, drive belt, bearing test module, and coupling. The bearing test module is installed with four bearings. Type and size of testing bearings are listed in Table 8. Five bearing health conditions (i.e., outer race fault (ORF), inner race fault (IRF), ball fault (BF), outer and inner race compound fault (OIRCF), outer race and ball compound fault (ORBCF)) on the normal bearing 1 are generated by using wire electrical discharge machining. Figure 13 shows the photos of different faulty bearings. In this experiment, the sampling frequency is set as 10,240 Hz, and motor speed is stable at 1050 rpm. In order to simulate the weak bearing fault signal brought by the long transmission path, one accelerometer is mounted on a position away from faulty bearing 1 to collect bearing vibration data of different health conditions. For each bearing health condition, 60 data samples with a length of 2048 points are obtained via the sliding window, where 30 data samples of each bearing health condition are randomly selected as the training set, and the other 30 data samples are regarded as the testing set. That is to say, the training set and the testing set each have 180 samples. Table 9 provides the detailed description of the experimental dataset. Figure 14 plots time domain waveforms of bearing vibration data under different health conditions in case 2. As shown in Figure 14, due to the environmental noise and transmission path interference, it is difficult to identify different bearing fault patterns by directly observing the time domain waveforms of bearing vibration signals, which means that it is urgent to adopt an effective method to identify different health conditions of rolling bearings.

**Figure 15:**
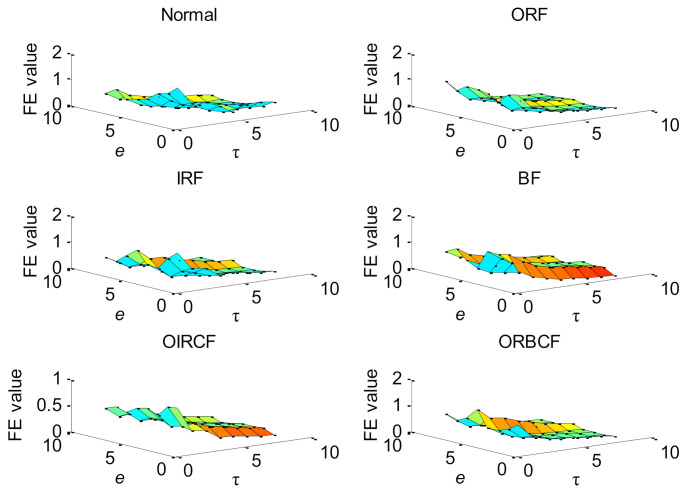
HMFE of bearing vibration data under different health conditions in case 2.

**Figure 16:**
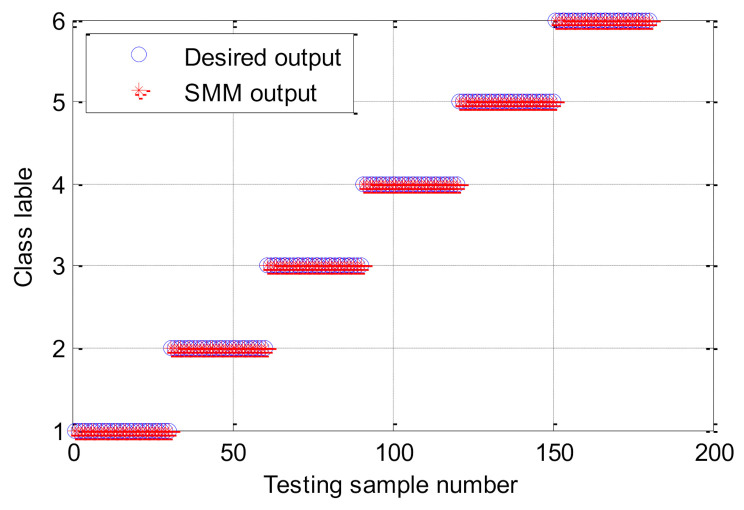
Classification results of the proposed method in case 2.

Firstly, to verify the effectiveness of the proposed method, the proposed method is utilized to analyze the experimental data listed in Table 9. According to the flowchart of the proposed method, the important parameters of HMFE are selected adaptively by using the BSA method. Concretely, in the HMFE method, the embedded dimension *m =* 4, the decomposition level *k* = 3, and the scale factor *τ* = 8 are used. Then, for each bearing health condition, the proposed HMFE method with the optimized parameters is conducted to obtain a multidimensional feature matrix with a size of 8 × 8 × 360. Figure 15 plots the calculation results of HMFE of one data sample of different bearing health conditions. Obviously, as seen from Figure 15, a comprehensive and recognizable feature matrix can be obtained by calculating HMFE. Finally, according to the sample percentage of 1:1, the obtained multidimensional feature matrix is randomly and averagely divided into the training sample matrix and testing sample matrix. The training sample matrix is adopted for the training of the SMM model, and the testing sample matrix is entered into the well-trained SMM model for identifying different bearing health conditions. Figure 16 gives the identification results of the proposed method in the first trial. It can be seen from Figure 16 that six bearing health conditions (i.e., Normal, ORF, IRF, BF, OIRCF, ORBCF) are all correctly identified by using the proposed method, which implies that the efficacy of the proposed method in bearing fault identification is preliminarily proven.

As with in case 1, to illustrate the validity and necessity of the parameter optimization process of the proposed method, we analyzed the identification results of the proposed method under different parameter settings, as listed in Table 10. Seen from Table 10, when the raw bearing vibration data are analyzed by the HMFE method with the optimal parameters (i.e., the embedded dimension *m =* 4, the decomposition level *k* = 3, and the scale factor *τ* = 8), the highest identification accuracy (100%) can be obtained, which means that the parameter selection of HMFE has a great influence on its diagnosis performance. Meanwhile, this also indicates that it is very useful for bearing fault identification by using BAS to select the important parameters of HMFE.

**Figure 17:**
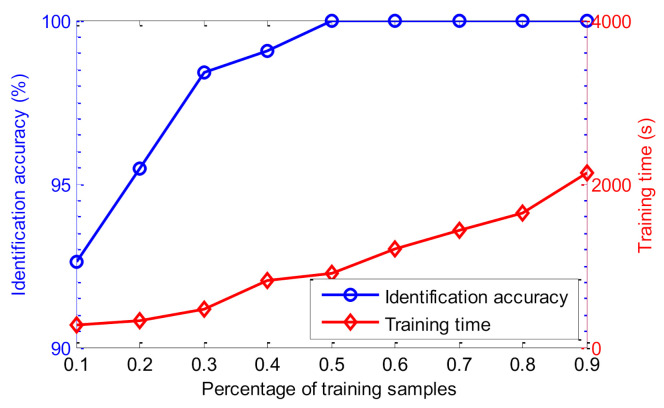
The identification results of the proposed method under different percentages of training samples in case 2.

Similarly, to show the influence of the number of training samples on the proposed method, the identification results of the proposed method at different percentage of training samples are considered. The identification accuracy and training time of the proposed method are calculated, and the calculation results are plotted in Figure 17, for when the percentage of the training samples compared to all samples is, respectively, 10% (36/360), 20% (72/360), 30% (108/360), 40% (144/360), 50% (180/360), 60% (216/360), 70% (252/360), 80% (288/360), and 90% (324/360). Seen from Figure 17, the identification accuracy and training time of the proposed method are increased with the increase of the percentage of the training samples. Therefore, in this paper, the percentage of training samples is set as 50% (i.e., the number of training and testing samples is the same) to achieve a compromise between the identification accuracy and training time, which is relatively reasonable.

To further verify the effectiveness of the SMM model used in the proposed method, the identification results of combining HMFE and different classifiers (i.e., SMM, SVM, ELM, and BPNN) are calculated, and the results are listed in Table 11. Note that the model parameters of different classifiers are set the same as case 1. It can be seen from Table 11 that the proposed method (i.e., the combination of HMFE and SMM) can achieve a higher identification accuracy compared with other combination methods. This further validates the effectiveness of using the SMM model in the proposed method.

**Figure 18:**
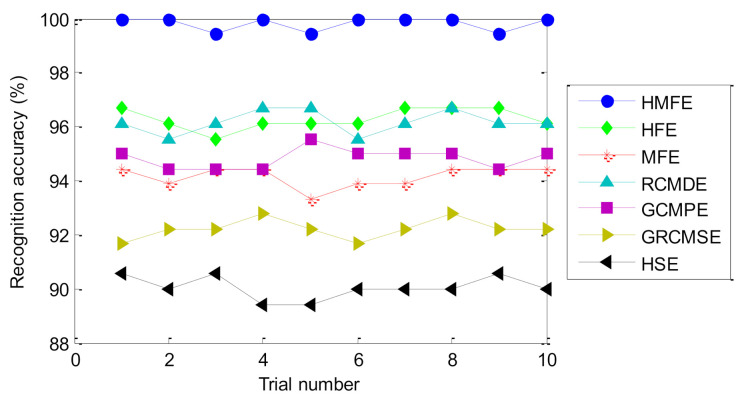
The identification results of 10 trials of different methods in case 2.

As in case 1, to further prove superiority of the proposed method, the proposed method and six entropies (i.e., HFE, MFE, RCMDE, GCMPE, GRCMSE, and HSE) are used to analyze the same experimental data. Again, 10 trials are performed to avoid the contingency of the diagnosis results, and the BSA method is used to determine the important parameters of all methods to ensure the fairness of the comparison. Table 12 gives the parameter settings of different methods. Figure 18 plots the identification accuracy of different methods over 10 trials. Moreover, Table 13 gives the specific comparison results of the identification accuracy of the different methods. Seen from Figure 18 and Table 13, compared with the other six representative methods (i.e., HFE, MFE, RCMDE, GCMPE, GRCMSE, and HSE), the proposed method can obtain the highest average identification accuracy (i.e., 99.83%), which further demonstrates the superiority of the proposed method for bearing health condition identification. Besides, it can be also found from Table 13 that the proposed method can obtain a smaller standard deviation (0.2705) than the other six representative methods (i.e., HFE, MFE, RCMDE, GCMPE, GRCMSE, and HSE), which implies that the proposed method has a more stable fault identification performance than the other six representative methods. Therefore, by the experiment and the comparative analysis, the superiority and efficacy of the proposed method in identifying bearing fault patterns are highlighted.

### 5.3. Further Discussion

Although the effectiveness and superiority of the proposed method in bearing health condition identification is verified by the above two experiments, some limitations and future works related to the proposed method are still left to discuss. These future works and limitations are summarized as follows:

(1) In the proposed method, the key parameters of HMFE are determined by the bird swarm algorithm (BSA) method, which is helpful for bearing fault feature extraction. We all know that some other advanced optimizer (e.g., Grey wolf optimization (GWO), Whale optimization algorithm (WOA), Grasshopper optimization algorithm (GOA)) can also be introduced to automatically determine the key parameters of HMFE. Therefore, in our future work, the parameter selection problem of HMFE will continue to be studied by adopting other advanced optimizers instead of the BSA method.
(2) In the final step of the proposed method, although the support matrix machine (SMM) is employed to achieve the automatic identification of fault patterns of rolling-element bearing and obtain a good diagnosis result, there are many other advanced classification models in the previously reported literature, including the improved versions (e.g., the non-parallel least squares support matrix machine [42], nonlinear kernel support matrix machine) of SMM and deep learning models (e.g., convolutional neural network [43], deep regularized variational autoencoder [44], deep belief network [45], and other deep learning methods [46]). Hence, in our future work, SMM of the proposed method will be replaced by these advanced classification models to automatically obtain the identification results of bearing fault patterns.
(3) The proposed method is proven to be effective for identifying the bearing health condition at constant speed, but it is unknown for bearing fault identification under variable speed. Hence, the proposed method will be extended to solve the problem of bearing fault identification under variable speed, which is regarded as our future focus. Besides this, in our future work, other faults (e.g., gear, rotor, and blade) of rotating machinery will also be diagnosed by applying the proposed method.
(4) In this paper, the proposed HMFE is only applied for single-channel sensor data analysis of rolling-element bearings. For bearing vibration data analysis of multi-channel sensors, with the help of the idea of multichannel data processing used in the existing multivariate multiscale entropy, a new multichannel data processing method named multivariate hierarchical multiscale fuzzy entropy (MHMFE) will be designed to solve the problem of multivariate fault diagnosis in our future work.
(5) Due to the addition of the parameter optimization algorithm, the biggest limitation of the proposed method lies in the large calculation time. Therefore, to solve this issue and improve the computational efficiency of the proposed method, in our future work, some sensitive indicators (e.g., Chebychev distance and Mahalanobis distance) can be used to instead of the complex optimizer to automatically select the key parameters of HMFE. Besides, in the running of the algorithm, graphic processing units (GPU) can be adopted instead of the central processing unit (CPU) to accelerate the calculation process of the proposed method.

## 6. Conclusions

This paper proposes a new intelligent bearing fault diagnosis method based on self-adaptive hierarchical multiscale fuzzy entropy, which can not only solve the disadvantages of artificial selection of important parameters of most existing entropies, but also obtain richer and more comprehensive bearing fault feature information. Two experimental cases validate the effectiveness of the proposed method in bearing fault identification. Furthermore, compared with some reported entropy methods, the superiority of the proposed method in bearing fault identification is verified. Some specific conclusions and contributions are summarized as follows:

(1) A new signal complexity metric method named hierarchical multiscale fuzzy entropy is developed by integrating the hierarchical decomposition into multiscale fuzzy entropy, which is aimed at improving fault feature extraction performance.
(2) An effective parameter optimizer called bird swarm algorithm is introduced to automatically choose several important parameters of hierarchical multiscale fuzzy entropy, which can avoid the dependence of parameter selection of the existing entropy on specialist experience.
(3) The effectiveness of the proposed method in the identification of bearing fault types and fault severity is verified by experimental and contrastive analysis. The experimental results show that compared with some existing representative multiscale entropies or hierarchical entropies, the proposed method can achieve broader and richer fault feature information and its identification accuracy has been greatly improved, which indicates that the proposed method has a certain competitiveness in bearing health condition identification. This study provides a new perspective for intelligent fault diagnosis for rolling-element bearings.

## Figures and Tables

**Table 1:** Performance comparison among different methods.

Methods	Euclidean Distance	Calculation Time (s)
HMFE	0.3421	5.167
HFE	0.2048	2.163
MFE	0.0613	9.768

**Table 2:** The specification of test bearing.

Bearing Type	Roller Diameter (mm)	Pitch Diameter (mm)	Number of the Roller	Contact Angle (°)
SKF6205-2RS	7.94	39.04	9	0

**Table 3:** The detailed description of experimental dataset in case 1.

Bearing Health Conditions	Abbreviation	Fault Size (inches)	Number of Training Samples	Number of Testing Samples	Class Label
Normal	NORM	0	25	25	1
Inner race slight fault	IRSF	0.007	25	25	2
Inner race medium fault	IRMF	0.014	25	25	3
Inner race heavy fault	IRHF	0.021	25	25	4
Outer race slight fault	ORSF	0.007	25	25	5
Outer race medium fault	ORMF	0.014	25	25	6
Outer race heavy fault	ORHF	0.021	25	25	7
Ball slight fault	BSF	0.007	25	25	8
Ball medium fault	BMF	0.014	25	25	9
Ball heavy fault	BHF	0.021	25	25	10

**Table 4:** The identification results of the proposed method with different parameters in case 1.

Parameter Setting of HMFE			Identification Accuracy (%)
The Embedded Dimension *m*	The Decomposition Level *k*	The Scale Factor *τ*	
1	2	4	87.60
2	2	6	93.20
2	3	7	96.80
3	3	7	98.80
3	3	8	100
4	3	8	99.20
4	4	12	98.40
5	4	16	98.80

**Table 5:** The identification results of combining HMFE and different classifiers in case 1.

Classifier	Identification Accuracy Obtained by Combining HMFE and Different Classifier Methods in 5 Trials					Average Accuracy (%)
	1	2	3	4	5	
SMM	100	99.60	100	100	100	99.92
SVM	97.60	98.00	97.60	97.60	97.20	97.60
ELM	98.40	98.80	98.40	98.00	98.40	98.40
BPNN	96.40	96.80	96.40	97.20	96.80	96.72

**Table 6:** Parameter settings for different methods in case 1.

Methods	The Optimal Parameter Setting
HMFE	The embedded dimension *m* = 3, the decomposition level *k* = 3, and the scale factor *τ* = 8, the similarity tolerance r=0.15×SD, where *SD* is the standard deviation of the original signal.
HFE	The embedded dimension *m* = 3, the decomposition level *k* = 3, the similarity tolerance r=0.15×SD, where *SD* is the standard deviation of the original signal.
MFE	The embedded dimension *m* = 3, the scale factor *τ* = 10, the similarity tolerance r=0.15×SD, where *SD* is the standard deviation of the original signal.
RCMDE	The embedded dimension *m* = 3, time delay *d* = 1, the number of classes *c* = 5, the scale factor *τ* = 10.
GCMPE	The embedded dimension *m* = 3, time delay *d* = 1, the scale factor *τ* = 12.
GRCMSE	The embedded dimension *m* = 3, the scale factor *τ* = 10, the similarity tolerance r=0.15×SD, where *SD* is the standard deviation of the original signal.
HSE	The embedded dimension *m* = 3, the decomposition level *k* = 3, the similarity tolerance r=0.15×SD, where *SD* is the standard deviation of the original signal.

**Table 7:** Comparison results of different methods in case 1.

Methods	Maximum Accuracy (%)	Minimum Accuracy (%)	Average Accuracy (%)	Standard Deviation
HMFE	100	99.60	99.92	0.1687
HFE	97.60	96.80	97.08	0.2700
MFE	96.00	95.20	95.44	0.3373
RCMDE	98.00	97.20	97.72	0.2700
GCMPE	96.80	96.00	96.20	0.2828
GRCMSE	94.80	94.00	94.28	0.3293
HSE	92.40	91.60	91.92	0.2530

**Table 8:** Parameters of rolling-element bearing.

Bearing Type	Ball Diameter (mm)	Pitch Diameter (mm)	Number of Balls	Contact Angle (°)
HRB6205	7.94	39.04	9	0

**Table 9:** Detailed description of the experimental dataset in case 2.

Bearing Health Conditions	Abbreviation	Number of Training Samples	Number of Testing Samples	Class Labels
Normal	NORM	30	30	1
Outer race fault	ORF	30	30	2
Inner race fault	IRF	30	30	3
Ball fault	BF	30	30	4
Outer and inner race compound fault	OIRCF	30	30	5
Outer race and ball compound fault	ORBCF	30	30	6

**Table 10:** The identification results of the proposed method with different parameters in case 2.

Parameter Setting of HMFE			Identification Accuracy (%)
The Embedded Dimension *m*	The Decomposition Level *k*	The Scale Factor *τ*	
1	2	4	89.44
2	2	6	93.33
2	3	7	97.22
3	3	7	98.33
3	3	8	99.44
4	3	8	100
4	4	12	98.33
5	4	16	98.89

**Table 11:** The identification results of combining HMFE and different classifiers in case 2.

Classifier	Identification Accuracy Obtained by Combining HMFE and Different Classifier Methods in 5 Trials					Average Accuracy (%)
	1	2	3	4	5	
SMM	100	100	99.44	100	99.44	99.77
SVM	97.22	96.67	97.22	96.67	96.11	96.78
ELM	98.33	97.78	98.33	97.22	98.33	97.99
BPNN	95.55	96.11	95.55	96.11	95.00	95.66

**Table 12:** Parameter settings for different methods in case 2.

Methods	The Optimal Parameter Setting
HMFE	The embedded dimension *m* = 4, the decomposition level *k* = 3, and the scale factor *τ* = 8, the similarity tolerance r=0.15×SD, where *SD* is the standard deviation of the original signal.
HFE	The embedded dimension *m* = 4, the decomposition level *k* = 3, the similarity tolerance r=0.15×SD, where *SD* is the standard deviation of the original signal.
MFE	The embedded dimension *m* = 3, the scale factor *τ* = 12, the similarity tolerance r=0.15×SD, where *SD* is the standard deviation of the original signal.
RCMDE	The embedded dimension *m* = 3, time delay *d* = 1, the number of classes *c* = 6, the scale factor *τ* = 12.
GCMPE	The embedded dimension *m* = 3, time delay *d* = 1, the scale factor *τ* = 15.
GRCMSE	The embedded dimension *m* = 4, the scale factor *τ* = 10, the similarity tolerance r=0.15×SD, where *SD* is the standard deviation of the original signal.
HSE	The embedded dimension *m* = 3, the decomposition level *k* = 3, the similarity tolerance r=0.15×SD, where *SD* is the standard deviation of the original signal.

**Table 13:** Comparison results of different methods in case 2.

Methods	Maximum Accuracy (%)	Minimum Accuracy (%)	Average Accuracy (%)	Standard Deviation
HMFE	100	99.44	99.83	0.2705
HFE	96.67	95.55	96.27	0.3780
MFE	94.44	93.33	94.16	0.3913
RCMDE	96.67	95.55	96.16	0.4132
GCMPE	95.55	94.44	94.83	0.3758
GRCMSE	92.77	91.67	92.22	0.3667
HSE	90.55	89.40	90.04	0.4226

## Data Availability

The data used in this study are all owned by the research group and will not be transmitted.
